# A non-reductive science of personality, character, and well-being must take the person's worldview into account

**DOI:** 10.3389/fpsyg.2014.00961

**Published:** 2014-08-28

**Authors:** Artur Nilsson

**Affiliations:** Department of Psychology, Lund UniversityLund, Sweden

**Keywords:** worldview, meaning-making, non-reductive materialism, personality, character, well-being

In his foundational work for personality psychology, Allport (1927, 1937) distinguished personality from character. Personality was, on Allport's account, a descriptive concept referring to a psycho-physical structure, whereas character was *personality evaluated* in accordance with moral norms. When he introduced the paradigmatic “lexical” method of deriving personality trait terms from the dictionary, he therefore sought to exclude all trait terms with ostensive normative content. This approach had a profound effect upon the field, and researchers are still today working on how to optimally purge personality of normative content (e.g., Bäckström et al., 2009; Pettersson and Turkheimer, 2010). Its appropriateness as a paradigm for the entire field of personality psychology can, however, be questioned (Kristjánsson, 2012; Nilsson, 2014). It is plausible that some personality characteristics particularly relevant to psychic illness, human flourishing, and moral behavior are intrinsically value-laden (Cloninger et al., 1993; Cawley et al., 2000; Peterson and Seligman, 2004).

I will focus on Cloninger's approach here, because he has, in addition to introducing an influential model of character, discussed the philosophical foundations of the study of character and well-being. For Cloninger (2004), character is not only value-laden; it refers to uniquely human aspects of personality representing “what people make of themselves intentionally” (p. 44), as contrasted with their animalistic *temperament*. He wants the science of character and well-being to transcend the dichotomy between materialist reductionism and Cartesian dualism, by taking the person's consciousness, agency, and processes of self-growth seriously while integrating this with knowledge about the human physical and biological constitution. Although I agree with this idea of having a *non-reductive* psychological science, I disagree with Cloninger about what it entails. I will therefore review Cloninger's (2004) approach from a philosophical perspective, in a critical and, hopefully, constructive way. I will defend a notion of non-reductive psychology based upon contemporary academic philosophy and argue that Cloninger's approach is not genuinely non-reductive. I will suggest that a non-reductive psychological science must take the person's worldview into account and argue that Cloninger's approach limits our understanding of human psychology by not considering the role of worldviews in the development of character and well-being.

## Non-reductive materialism

Today, philosophers who seek to transcend the dichotomy between reductive materialism and Cartesian dualism generally adopt some version of *non-reductive materialism* (Davidson, 1963, 1970; Fodor, 1974; Searle, 1983, 1992; Chalmers, 1996), claiming that although all mental states and events are causally realized in the brain, there is not a particular *type* of brain state corresponding to each *type* of mental state. The reason for this is that we identify and individuate mental states in terms of a folk psychological language of “attitudes,” “beliefs,” “desires,” “emotions,” “goals,” etc., which is *holistic*, insofar as it describes mental states as partly constituted by their relations to each other and their neurophysiological realization and behavioral manifestation as therefore dependent upon the entire network of mental states. In other words, on non-reductive materialism, no particular belief, goal, desire, or other intentional state, let alone a more complex folk psychological concept such as “personality,” “character,” or “well-being,” can even in principle be isolated and *reduced* to neurophysiology or behavior, and these irreducible folk psychological concepts are crucial for understanding human psychology.

A key implication of non-reductive materialism is that human experiences and actions are imbued with meaning; to treat human beings as *persons*, rather than mere mechanical systems or animals, is to treat them as linguistic beings, who construct reasons and act upon them (Hacker, 2007), partly driven by needs to create and sustain meanings and to assuage fears and anxieties fueled by their uniquely human awareness of their existential condition (Nilsson, 2013). Although meaning-making is today studied in such different fields as the psychology of adaptation and well-being (Janoff-Bulman, 1992; Wong, 2012), social psychology (Greenberg et al., 1986; Heine et al., 2006), and neuropsychology (Gazzaniga, 2005), researchers rarely take into consideration the fact that meaning is constructed within a *worldview*—the person's most basic beliefs, values, constructs, and scripts for understanding, evaluating, and acting upon reality, which ground the network within which more specific beliefs, goals, intentions, etc., are embedded. A person necessarily lives *through* a worldview—s/he can only, for example, act, morally or immorally, upon a worldview, and experience well-being, in its distinctly human form, through a worldview. A non-reductive psychological science must therefore treat the person's worldview as an aspect of personality *in its own right*, not reducible to behavioral or mental regularities (i.e., traits; Nilsson, 2014). Although personalists (Allport, 1937; Stern, 1938; Mounier, 1952; Lamiell, 1987), narrative psychologists (Tomkins, 1965, 1979; McAdams, 1992, 2008), and construct psychologists (Kelly, 1955; Little, 2005) have contributed to such an endeavor, worldviews do not receive the attention they deserve in contemporary psychology (Koltko-Rivera, 2004; Nilsson, 2013, 2014).

## Cloninger's transcendentalism

Cloninger's(2004) approach instead merges elements of folk spirituality (cf. Forman, 2004), Eastern thought, Hegelian metaphysics, and quantum physics. He suggests that a person's consciousness can be developed, through a process catalyzed by meditation, reflection, and contemplation, toward increasing self-awareness, wisdom, goodness, and well-being. In the final, *self-transcendent* stage, the person is freed of all “dualistic” thought of body, mind, and spirit as separate and recognizes that “the individual mind is like a node in a universal Internet of consciousness” (p. 36), thereby attaining “coherence” of body, mind, and spirit, unconditional well-being, potential access to other minds, and “direct self-aware perception of what is real and true without misunderstanding as a result of preconceptions, prejudices, fears, desires, and conflicts” (p. 325). Cloninger (2004) also draws parallels between self-transcendent consciousness and quantum phenomena, including the impossibility of precisely determining the state and location of quantum particles (“*non-locality*”) and the Higgs field within which particles acquire mass, and he claims, furthermore, that the unpredictability (“*non-causality*”) of quantum physical events is “another way of talking about freedom” (p. 73) and that “the thought of gifted people involves intuitive leaps or quantum jumps, not deductive algorithms” (p. 65; cf. Capra, 1975).

Cloninger (2004, p. 317) makes clear that what he is proposing is not just a psychological theory, but also a philosophy of science:

The science of well-being is founded on the understanding that there is an indissoluble unity to all that is or can be. The universal unity of being is recognized widely as an empirical fact, as well as an essential organizing principle for any adequate science [..] the universal unity of being is the only viewpoint consistent with any coherent and testable science.

This passage is puzzling insofar as it describes the postulated unity of being both as empirical fact, which implies that it is open to empirical refutation, and as *essential* organizing principle constitutive of research in this area, which implies that it is, in Quine's (1953) terminology, close to the center of the scientific field and therefore not easily changed. Given that Cloninger (2004) suggests that recognition of the unity of being-thesis is ultimately intuitive and not amenable to rational argumentation or objective test, and that its critics lack self-awareness, this thesis is more properly treated as a presupposition and interpretive framework than as an empirical fact (Popper, 1959).

But whether this is an appropriate, non-reductive foundation for the study of persons is questionable. On the non-reductive account I am proposing, what is *essential* is that we take the person's subjective experiences and their meanings seriously, *in psychological terms*, treating them as *real and irreducible*; not that we assume that special forms of experience convey true insight into the nature of reality. One problem with Cloninger's approach is precisely that it does not give meaning-making the role that it deserves in personality measurement and explanation of experience and action. Cloninger (2004) offers parallels to quantum physics rather than an account of reason-based explanation (Davidson, 1963; Searle, 1983) and Cloninger et al. (1993) measure character with traditional trait-type items which focus on typical behaviors and experiences, rather than worldview-type items which ask persons about their most basic beliefs, values, goals, and so on (Nilsson, 2014). Cloninger's use of quantum physics to describe the mind is, furthermore, whether interpreted as an “analogy” (p. 65) or as an explanation of “actual” processes underlying self-aware consciousness (p. 328), difficult to reconcile with non-reductive materialism. Although it is conceivable that the hitherto unidentified mechanisms through which the brain causes consciousness, agency, and certain qualitative feels operate at the quantum level (Chalmers, 1996; Searle, 1997), the folk psychological concepts that render our experiences and actions meaningful and agentic are, because of their *logical* holism, as irreducible to quantum physics as to classical physics, and we have little reason to assume that the *causes* of conscious experiences are isomorphic with their qualitative feels (Stenger, 1993; cf. Brown et al., 2013). Similar to this, Cloninger's (p. 38) invocation of Allport's definition of personality as a “psycho-physical system” is inconsistent with non-reductive materialism, insofar as it is understood as implying that personality can be reduced to a neuro-physiological causal system (Nilsson, 2013). Finally, the Hegelian monist metaphysics Cloninger (2004) draws upon is rejected today even by Hegelians. For example, Pippin (1989, p. 4)—one of several philosophers reinterpreting Hegel in non-metaphysical terms *in order to rehabilitate his philosophy*—thinks that the “metaphysical monist or speculative, contradiction-embracing logician [..] is not the historically influential Hegel.”

## Implications for research

Cloninger et al. (1993) model divides character into: (1) *self-directedness*, or agency, which incorporates acting deliberatively on personal goals and values, taking responsibility for actions, and developing resources for goal pursuit and self-acceptance, (2) *cooperativeness*, or communion, which incorporates compassion, empathy, helpfulness, acceptance of others, and acting on moral principles rather than self-interest, and (3) *self-transcendence*, which incorporates a sense of unity underlying the universe and connecting the self with the world around it, intuitive apprehension of relationships that cannot be explained rationally or observed objectively, and experiences of flow, absorption, and self-forgetfulness. These aspects of character correspond, respectively, to the person's relation to the self, to others, and to the universe. As such, they undoubtedly refer to basic aspects of our intentional engagement with the world. But the model does not take different worldviews into account. Self-transcendence, in particular, appears conflated with *spiritual self-transcendence*—that is, self-transcendence *through* spirituality. Self-transcendence, in a more general sense, can be understood as the pursuit of meaning and identity through participation in, and selfless contribution to, something larger than the self, whether this is a divine or spiritual reality, a community of persons or sentient beings, or an ideological ideal (Schwartz, 1992; MacDonald et al., 1998; Koltko-Rivera, 2004). It requires only that the person is connected to the outside world through intentional directedness at, and engagement with, that world; it does not require an actual physical or spiritual connection between the person and that toward which s/he directs him-/herself.

More generally, I suggest that character can be understood in terms of the interaction between the three proposed dimensions and the person's worldview, and that researchers therefore need to investigate how different worldviews facilitate and inhibit the development of character. Because character is an intrinsically normative concept, what counts as character is partly an empirical question—character is what turns out to produce desirable psychological, moral, and social consequences. We might ask, for example, if, and if so *how*, different worldviews can be reconciled with *ethical* self-transcendence, selfless love, genuine happiness, tolerance, creativity, autonomy, and experiences of wonder, beauty, and awe. It is, I suggest, unlikely that there is one ultimate path of character development suitable for all persons. Cloninger's (2004, p. 29) own observation that “outstanding exponents of positive philosophy have often had limited success in helping their followers develop coherence” is true, I suggest, partly because neither worldview nor the development of character and well-being is a one-size-fits-all. By considering the full potential range of personalities emerging from the diversity of human worldviews, we can, I contend, better understand and encourage the development of character and well-being, thus potentially harnessing the full positive potentials of humanity for cultural and social progress (cf. Cloninger, 2004, 2008, 2013).

### Conflict of interest statement

The author declares that the research was conducted in the absence of any commercial or financial relationships that could be construed as a potential conflict of interest.
